# Cost-effectiveness and cost-utility of the ball-and-socket trapeziometacarpal prosthesis compared to trapeziectomy and ligament reconstruction: study protocol for a randomized controlled clinical trial

**DOI:** 10.1186/s13063-024-08057-1

**Published:** 2024-03-27

**Authors:** Serafín Lirola-Palmero, Guillem Salva-Coll, Aina María Yáñez-Juan, Eduardo Sánchez-Iriso

**Affiliations:** 1 Departmen of Hand Surgery and Microsurgery, Hospital Universitari Son Espases, Ctra. Valldemosa 79, Palma de Mallorca, 07120 Spain; 2 University of the Balearic Islands, Palma de Mallorca, Spain; 3 Universidad Pública de Navarra, Pamplona, Spain

**Keywords:** Trapeziometacarpal, Osteoarthritis, Arthroplasty, Trapeziectomy, Ball-and-socket prosthesis

## Abstract

**Background:**

Trapeziometacarpal (TMC) osteoarthritis (OA) is a common cause of pain and weakness during thumb pinch leading to disability. There is no consensus about the best surgical treatment in unresponsive cases. The treatment is associated with costs and the recovery may take up to 1 year after surgery depending on the procedure. No randomized controlled trials have been conducted comparing ball and socket TMC prosthesis to trapeziectomy with ligament reconstruction.

**Methods:**

A randomized, blinded, parallel-group superiority clinical trial comparing trapeziectomy with abductor pollicis longus (APL) arthroplasty and prosthetic replacement with Maïa® prosthesis. Patients, 18 years old and older, with a clinical diagnosis of unilateral or bilateral TMC OA who fulfill the trial’s eligibility criteria will be invited to participate. The diagnosis will be made by experienced hand surgeons based on symptoms, clinical history, physical examination, and complementary imaging tests.

A total of 106 patients who provide informed consent will be randomly assigned to treatment with APL arthroplasty and prosthetic replacement with Maïa® prosthesis. The participants will complete different questionnaires including EuroQuol 5D-5L (EQ-5D-5L), the Quick DASH, and the Patient Rated Wrist Evaluation (PRWE) at baseline, at 6 weeks, and 3, 6, 12, 24, 36, 48, and 60 months after surgical treatment. The participants will undergo physical examination, range of motion assessment, and strength measure every appointment. The trial’s primary outcome variable is the change in the visual analog scale (VAS) from baseline to 12 months. A long-term follow-up analysis will be performed every year for 5 years to assess chronic changes and prosthesis survival rate. The costs will be calculated from the provider’s and society perspective using direct and indirect medical costs.

**Discussion:**

This is the first randomized study that investigates the effectiveness and cost-utility of trapeziectomy and ligament reconstruction arthroplasty and Maïa prosthesis. We expect the findings from this trial to lead to new insights into the surgical approach to TMC OA.

**Trial registration:**

ClinicalTrials.gov NCT04562753. Registered on June 15, 2020.

**Supplementary Information:**

The online version contains supplementary material available at 10.1186/s13063-024-08057-1.

## Background

The prevalence of trapeziometacarpal (TMC) osteoarthritis (OA) is about 30% of people older than 50 years old, more common in women between 50 and 70 years old [1–3].

Symptoms are variable ranging from pain and swelling in the base of the thumb to weakness during thumb pinch. All of them lead to difficulties in daily living activities reducing capacity to work, especially in those that require repetitive movements of fist and clamp closure, impairing the quality of life and general health [3–6].

There is no consensus on the best treatment for TMC OA. Surgical approach is often considered in severe or unresponsive cases to conservative treatment. Numerous surgical alternatives have been described but the evidence still fails to conclusively show superiority among options [4–6]. Surgical indication preference is based on personal experience rather than published clinical evidence [4, 7, 8].

The most commonly used surgical treatment is trapeziectomy with or without ligamentoplasty and/or tendon interposition [9–12]. Patients achieve good results in terms of pain relief and movement in 85% of the cases, but without a significant gain in strength [13]. Recovery time in these procedures may take up to 1 year after the surgical procedure [14, 15]. The development of ball-and-socket prosthetic designs has been based on the premises of maintaining mobility, preventing proximal thumb migration, and improving pinch strength with faster recovery compared to other procedures [3, 14, 16, 17].

Nineteen different designs of prostheses have been described since 1979 [17]. Cebrián-Gomez et al. showed better results in abduction, adduction, compression strength, improvement in pain, Quick Dash test, patient satisfaction, and return to daily activities using Maïa TMC prosthesis compared to trapeziectomy with ligament reconstruction and tendon interposition [18]. Martin-Ferrero showed a 93% survival rate at ten years for ARPE prosthesis [19, 20], a similar design to Maïa® prosthesis [21]. A total joint replacement should be significantly better than trapeziectomy or its modifications to justify its use because of the added cost of the implant [17].

Based on this background, and taking into account the lack of evidence, we have designed a prospective randomized clinical trial to compare the clinical results, safety, and cost-utility of implant arthroplasty with the Maïa® prosthesis compared to trapeziectomy with abductor pollicis longus (APL) arthroplasty in the surgical treatment for TMC OA.

## Methods

### Objective

The primary objective of the trial is to investigate whether an arthroplasty implant is clinically more effective in reducing symptoms and improving function compared to trapeziectomy with ligament reconstruction arthroplasty in patients with TMC OA unresponsive to conservative treatment.

The secondary objectives are to determine the cost-effectiveness and cost-utility of implant arthroplasty compared to trapeziectomy with APL arthroplasty, from a National Healthcare System (NHS) and society perspective. In the longer term, we will evaluate differences in symptoms, function, radiological changes, work absence, and other health care resources in both surgical treatments for TMC OA.

### Design

The study is a single-center, prospective randomized, blinded, parallel-group superiority clinical trial comparing trapeziectomy with APL arthroplasty and arthroplasty with Maïa® prosthesis in patients with TMC OA. The results will be reported following the CONSORT (Consolidated Standards of Reporting Trials) statements [22]. The study will be conducted at the Department of Hand Surgery, Hospital Son Espases, Spain. The Standard Protocol Items Recommendations for Interventional Trials (SPIRIT) checklist was applied [23]. Ethical approval was obtained from the Ethical Committee (IB4144/20PS).

### Eligibility criteria

The eligibility criteria (Table 1) were designed to select a relatively homogeneous group of patients with TMC OA, suitable for either implant arthroplasty or trapeziectomy with APL arthroplasty.

**Table 1:** Eligibility criteria

Inclusion criteria	Exclusion criteria
Male or female ≥ 18 years	CTS clinical findings
A clinical diagnosis of unilateral or bilateral TMC OA	De Quervain’s tendinosis in the hand to study
Unresponsive cases to conservative treatment for 3 months [24]	Any previous surgery on the affected wrist/hand to study
Eaton classification	Rheumatoid arthritis
- Stage 2	
- Stage 3	
Written informed consent provided by the patient, prior to any trial-specific procedures	Trigger Finger in the hand to study
	Clinical suspicion of local or systemic sepsis or infection
	Current or previous infection of the affected hand
	Pregnant or lactating females
	Allergy to any of the implant materials
	Known abuse of drugs or alcohol
	Involved in on-going litigation cases for their condition
	Arthritis, chondrocalcinosis

For participants with bilateral TMC OA, a study hand will be designated by the participant based on the most severe symptoms. Patients will not be allowed to enter the trial more than once.

#### Inclusion criteria

The study population will consist of adults aged 18 years and over with a clinical diagnosis of unilateral or bilateral TMC OA that failed to improve with conservative treatment including splint, for at least 3 months [24–26].

The diagnosis will be made by an experienced hand surgeon based on the presenting symptoms, clinical history, physical examination, and complementary imaging tests [27, 28].

TMC OA is defined radiologically by the Eaton Classification. Only grades 2 and 3 will be included in the study. Slight carpometacarpal joint space narrowing, sclerotic changes, and osteophytes < 2 mm on the dorsal or volar side of the trapezium define stage 2. Stage 3 includes joint space markedly narrowed or obliterated, cystic changes, sclerotic bone, osteophytes > 2 mm, and normal scaphotrapezial (STT) joint [28–30].

#### Exclusion criteria

Participants with any of the following diagnoses will be ineligible for the trial: carpal tunnel syndrome (CTS) symptoms, De Quervain’s tenosynovitis, rheumatoid arthritis, trigger finger, or previous surgery on the hand to study. Other exclusion criteria are listed in Table 1.

### Recruitment, screening process, and enrolment

**Fig. 1:**
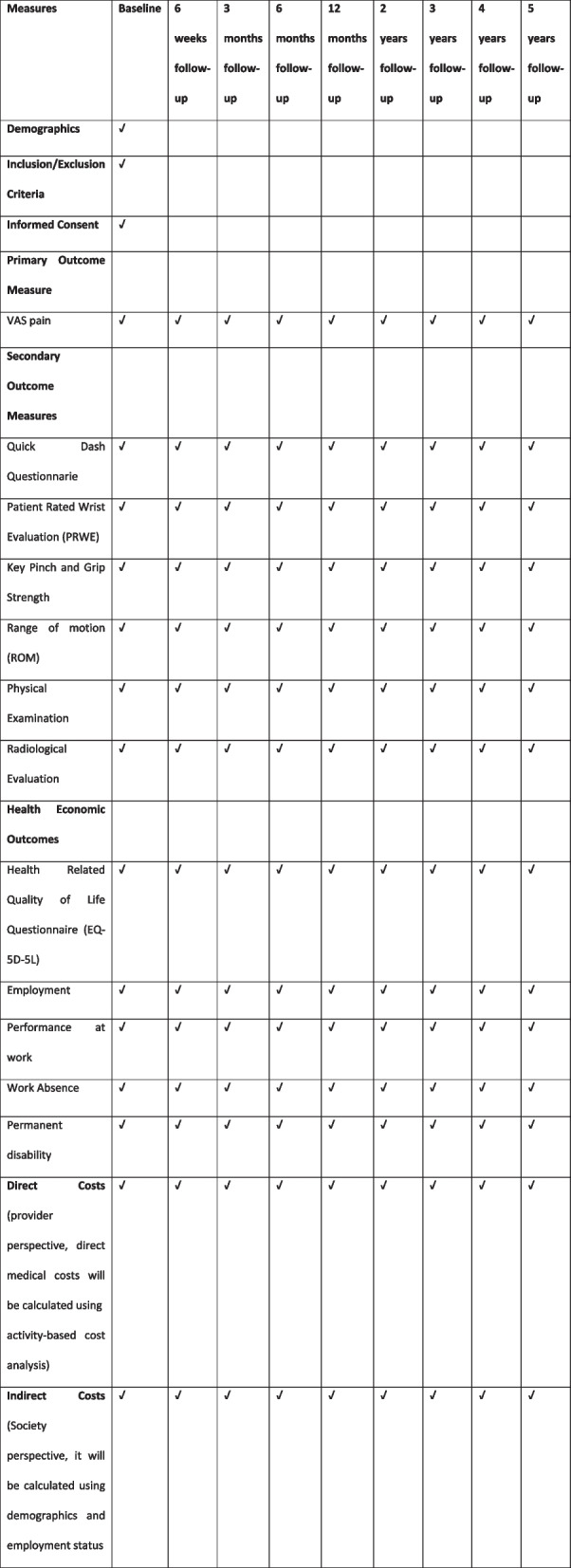
Trial timeline

Figure 1 describes the trial timeline. The patients referred to the hand surgery office for symptoms suggestive of TMC OA will be screened and assessed. Patients diagnosed with TMC OA will use a thumb abduction splint for 3 months, the first 3 weeks 24 h a day, and then at night for up to 3 months together with ergonomic measures. Unresponsive cases to conservative treatment and stage two or three of the Eaton radiological classification will be proposed to enroll.

Patients will be informed verbally about the trial and will be provided with an information report. The senior surgeon will answer any questions and the patient will be required to provide full written consent.

All participants will complete the baseline questionnaire prior to randomization. It includes questions regarding demographic characteristics, outcome measures, and potential prognostic factors.

### Recruitment strategy

Patients are referred to the Hand Surgery Unit by primary care physicians. All patients with thumb base osteoarthritis are evaluated by specialists in the Hand Surgery Unit of the Hospital. They are individually assessed to determine if they meet the inclusion criteria for the clinical trial. If they meet the criteria, the senior surgeon, after explaining the study, offers them all the possibility to enroll in the study. Since it is a single-center trial, patients from other centers or areas cannot be recruited unless the patient themselves requests to be seen and treated at our hospital, or is referred by another specialist. No financial or non-financial incentives are provided to the trial participants. Recruitment is expected to be completed in 5 to 6 years.

### Randomization and blinding

Patients will be randomized by the senior hand surgeon (Dr. Salva-Coll), via a remote web-based randomization system (OxMar Software, *Oxford Minimization and Randomization, an open source software*) [31], which ensures a concealed allocation sequence. Randomization will be stratified by sex and group age. Each participant will be assigned a unique identification number and a unique data case report form.

The participants and the senior hand surgeon will know the assigned treatment. The participants will have the appointments and controls as in our usual clinical practice.

The main investigator will be blinded to the treatment assignment and will collect the measures in the collection notebook, except for the radiological variables because of their association with the participant’s ID. The main investigator will assess the participant before the surgeon, on the same day in a different office.

### Operative treatment

Participants will be included in a protocol of major outpatient surgery under regional or general anesthesia depending on the anesthesiologist. Preoperative cefazolin (2 g) or vancomycin 1 g in case of allergy will be administered.

#### Group A: trapeziectomy and ligamentoplasty with abductor pollicis longus (APL) and tendon interposition

Participants randomized to Tendon Interposition Arthroplasty will undergo the technique described by Wulle [32] with a modification in the incision design:

1. TMC approach through a dorsal V-shaped skin incision. Sharp dissection of the subcutaneous tissue down to the joint’s capsule. Careful dissection and protection of the sensory branches of the radial nerve.
2. Approach to the joint’s capsule. At the radial border of the wound, the extensor pollicis brevis (EPB) tendon can be identified. In the proximal corner of the wound, the radial artery should be dissected and protected. The capsule is opened longitudinally between EPB and EPL with sharp dissection of all connecting ligaments along the trapezium.
3. Excision of the trapezium. The bone can be excised in a piecemeal fashion removing the fragments with a rongeur, while protecting the flexor carpi radialis (FCR) tendon.
4. Longitudinal split of the abductor pollicis longus (APL) tendon. Transection of the ulnar half of the APL tendon proximally as far as the incision allows.
5. The abductor pollicis longus (APL) tendon slip is wrapped around the flexor carpi radialis (FCR) tendon from radial to ulnar using a hemostat.
6. With the flexor carpi radialis (FCR) tendon pulled in a radial and proximal direction, the tendon slip is sutured to the FCR tendon using 3/0 non-resorbable sutures.
7. The remaining strip of the abductor pollicis longus (APL) tendon is passed through the remnant of the dorsoulnar capsule sutured and back to the FCR. The rest of the APL is inserted between the metacarpal and the scaphoid to act as a spacer.
8. Closure of the joint capsule using 4/0 resorbable suture. Closure in layers. Compressive bandage with a splint in thumb abduction on the first web space. Release of the pneumatic tourniquet.

#### Group B: joint prosthesis

Participants randomized to Joint Prosthesis Arthroplasty with Maïa® (Groupe Lépine ™) will undergo the standard technique described in the literature [14] and the surgical technique provided by Groupe Lépine (implant designer and distributor) for the implant with a modification in the incision design:

1. TMC approach through a dorsal V-shaped skin incision. Sharp dissection of the subcutaneous tissue down to the joint’s capsule. Careful dissection and protection of the sensory branches of the radial nerve.
2. Approach to the joint’s capsule. At the radial border of the wound, the extensor pollicis brevis (EPB) tendon can be identified. In the proximal corner of the wound, the radial artery should be dissected and protected. Capsule opening using a longitudinal incision.
3. First metacarpal osteotomy. Parallel osteotomy 5 mm proximal of the base, oblique volar osteotomy to remove the volar osteophytes and to allow correct mobilization of the metacarpal to expose the trapezium. The intramedullary canal of the first metacarpal is reamed until stable bone contact is achieved. The trial metacarpal implant is placed and the correct size is confirmed radiologically. The trial is removed and the definitive metacarpal implant is placed.
4. Resection of medial osteophyte of the trapezium and trapezial cup prosthesis placement. Medial and lateral osteophytes are removed. The middle area of the trapezium is located with an awl and verified with a fluoroscope. The trapezium is reamed with the hemispherical specifically designed burrs. The definitive component is placed, supervising that it has correct bone coverage.
5. The neck longitude is determined through different trials, stability is verified and the final component is placed.
6. Radiologic control and closure in layers. Compressive bandage with a splint in thumb abduction on the first web space.

### Post-operative care

All patients will be included in a same day outpatient surgery protocol, and discharged from the hospital few hours after the surgery. The post-operative pain management is a combination of ibuprofen 600 mg/12 h, paracetamol 1 g/8 h, tramadol 50 mg/8 h and omeprazole 20 mg every 24 h, taking into account the possible adverse effects or contraindications.

All participants will be asked to attend to the first appointment with a nurse 2 days after surgery, to check the wound and to change the bandage. Patients will be asked about the tolerability of the surgery and any adverse events will be recorded. The next appointment will be about 10 days later to remove stitches, maintaining the splint until 3 weeks after the surgery.

Group A participants must wear a removable abduction splint, for 3 more weeks. The patients will be instructed on how to put and remove the abduction splint to start opposition exercises. Finally, Group A participants will start a standardized rehabilitation protocol.

Group B participants the splint will be removed 3 weeks after surgery. They will be instructed on how to do opposition and strengthening exercises at home. They will not attend to rehabilitation sessions in the hospital.

### Adverse events

During the trial, participants will be asked about adverse events. All adverse events will be reported and followed up until solved or as required. The type and duration will be recorded.

Participants will be asked to contact the trial investigator or hand surgeon whenever they wish to discuss or report any events during the trial. Concomitant cares are not permitted.

### Follow-up and outcomes measures

Patients will complete a baseline questionnaire, which includes clinical, sociodemographic data, and potential prognostic factors.

The primary outcome measure will be the visual analog scale (VAS) determined by changes in the distance between the patient-made marks. VAS is reliable, valid, and responsive in detecting improvements associated with pain treatment [33].

Secondary outcome measures include the Quick Disabilities of Arm, Shoulder, and Hand (QUICK DASH), a short, reliable, and valid measure of physical function and symptoms related to upper-limb musculoskeletal disorders [34, 35]. Patient-Rated Wrist Evaluation (PRWE) is a widely used measure of patient-reported disability and pain related to wrist disorders [36]. The grip strength using a dynamometer [37], followed by lateral (key) and pulp-to-pulp pinch, will be recorded (both in kilograms) with a JAMAR pinch meter [38]. The goniometry measurement is determined by a change in the range of motion, as defined by the IFSSH (Atlas of Surface Anatomy and Joint Motion. International Federation of Societies for Surgery of the Hand [Internet].Illinois. Accessed 18 December 2018. < https://ifssh.info/terminology_hand_surgery.php >). The health-related quality of life questionnaire EuroQol 5-dimensions (EQ-5D-5L) is a standardized instrument for measuring generic health status [39].

Health economic outcomes include the impact of the surgery on work and other activities (including work absence and reduction in work permanence). Direct medical costs will be calculated using activity-based cost analysis. Indirect costs will be added to the direct costs, which will be calculated using the demographics variables. The remuneration (average earnings or salary) is considered a reasonable measure of labor productivity.

The main investigator will check that the questionnaires are complete. If participants choose to withdraw from the trial, they will be asked if they agree with their data being used in this study.

### Data collection method and management

Before surgery, every measure has to be collected in baseline data form. If not collected the patient could not be operated on until it is done. Then after surgery, every appointment is duplicated, with the hand surgeon and principal investigator separately. If the patient does not attend the appointment, will be called by phone with a new appointment.

Each participant will be assigned a unique identifier and a unique data case report form that includes all measures collected at the different time-points (Fig. 1) and will be used for all data documentation to assure the participant’s confidentiality. All data will be digitalized and stored in a central database. Data will be stored for at least ten years after study completion.

The senior hand surgeon will keep all records related to randomly assigned participants. The trial will be monitored regularly and the records will be examined on a monthly basis by two members (principal investigator and a statistician), to ensure that the conduct of the trial is in accordance with the trial protocol. The steering committee will comprise the principal investigator, a statistician, and a senior hand surgeon. The data management team will include the trial’s principal investigator (SL) and a statistician (AY). A follow report will be reported to the Ethics Committee every year.

The full protocol and dataset supporting the findings of the study will be available in the Docusalut repository (https://docusalut.com). Results will be presented at national and international meetings, and published in peer-reviewed orthopedics, hand surgery, and hand therapy journals.

### Statistical analysis

#### Sample size

A total of 106 participants will be necessary (53 subjects for each treatment group) to detect differences equal to or greater than 30% in the visual analog scale (VAS) on the baseline status of the patient [33]. Accepted values will be for an alpha risk of 0.05 and a beta risk of 0.1, in a bilateral contrast. The common standard deviation is assumed to be 1.5. It is assumed that 10% of the trial patients will be lost.

#### Primary analysis at 1 year

The results will be analyzed by using Statistical Package for Social Sciences (SPSS 20.0, IBM Corp., Armonk, NY, USA) software. A descriptive analysis of the collected data will be obtained by using the mean and the standard deviation for the quantitative variables that follow a normal distribution. For those whose data do not follow normal distribution, the median and range will be calculated.

Differences shall be detected by chi-square or Fisher’s tests for categorical variables and Student’s *T* or Mann–Whitney test for the resultant variables. After randomization, a descriptive comparison will be carried out to find out similar variable distribution.

The primary analysis will be carried out by intention-to-treat (ITT). The main variable, VAS, will be used as a continuous variable to confirm the main hypothesis, and a non-parametric mean comparison analysis (Mann–Whitney *U* test) will be performed.

The clinical effectiveness analysis will follow the recommendations established by CONSORT, including its safety extension [40].

### Secondary analysis

Similar analysis will be performed every year until 5 years to evaluate the evolution of both surgical treatments and their implication on daily living activities. Moreover, to perform a long-term follow-up to assess the survivorship of the prosthesis or secondary changes in the APL arthroplasty group.

#### Health economics

From the provider’s perspective, direct medical costs will be calculated using activity-based costing analysis [41].

From the society perspective, indirect costs will be added to the direct costs. Indirect costs will be calculated using demographics variables (age, sex, educational level, employment status, and profession). It is based on the human capital models applied to the field of health [42]. According to this approach, the remuneration in the labor market (salary) is considered a reasonable measure of labor productivity. Based on this, the salaries that the participant ceases to receive will be estimated if they leave the job due to illness, temporarily or permanently [43, 44]. The loss of work will be calculated through the gross salary per work day (adjusting for age and sex), using data from the Salary Structure Survey of the Balearic Islands.

The value of the work performed by people in a situation of reduced productivity (“presenteism”) will be calculated as part of the sensitivity analysis, taking into account an assessment of the loss of productivity estimated by the patient and adjustment made using EQ-5D-5L [45].

The effectiveness will be based on the changes between preoperative and 1-year postoperative assessments. Incremental cost-effectiveness ratios (ICER) between both interventions will be estimated 1 year after surgery.

The cost-utility analysis will be based on the Quality Adjusted Live Year (QALY) like utility measure. Spanish version of EQ-5D-5L will provide the utilities. The incremental cost-utility ratio (ICUR) will be estimated as the ratio between the costs and QALY differences [46].

The same analysis will be performed at 1, 2, and 5 years to assess the evolution of the treatment.

#### Ethics

The Balearic Island Ethical Committee has approved the trial (reference number: IB4144/20. Date: July 20, 2020).

## Discussion

Trapeziometacarpal Osteoarthritis is a common condition that causes disability. The symptoms could lead to difficulties with daily living activities and reduce capacity to work, impacting quality of life and general health [3–5]. Conservative treatment could be helpful; however, if failed, surgical treatment is considered a definitive solution [4, 6].

Many surgical procedures have been described, with preferences frequently based on personal experience [4, 7, 8]. Current trends in surgical treatment suggest trapeziectomy and ligament reconstruction arthroplasty are the most popular treatments among hand surgeons [9, 11, 12].

Several reviews have been published, all of them evaluating surgical alternatives with similar results. In conclusion, neither treatment shows superiority over others. However, trapeziectomy alone demonstrated lesser complications in contrast with the rest of the alternatives. Despite the evidence showing no benefit to ligament reconstruction after trapeziectomy, 93% of American hand surgeons prefer it over other surgical procedures [47]. Wajon updated his reviews in 2009 and 2015 and showed superiority in range of motion for trapeziectomy and ligament arthroplasty [4, 6, 8]. The lack of long-term follow-up for trapeziectomy alone could be the reason for the surgeon’s preference [6, 12, 13]. Taking into account the literature, the evidence for the treatment of TMC OA is not strong enough to change the clinical practice.

The effort to maintain range of motion and improve pinch strength with lesser recovery time has been the basis for developing different implants. However, none of them have achieved wide acceptance [16, 17]. Since 1979, the popularity of ball and socket prostheses has grown due to improvements in the design and survival curve at 10 years up to 93% [19, 20]. In addition, recent studies have shown better results for range of motion, Quick Dash, pain relief, patient satisfaction, pinch strength, and recovery time [19, 20, 48]. The most common complication of the prosthesis is related to dislocation and loosening of the components that may require revision surgery of the implant. Toffoli et al. observed 5 Maïa prosthesis failures over 80 patients (5.2%) [21], Bricout et al. reported 18 surgical revisions performed out of 156 patients [49] and Martin-Ferrero estimated a survivorship for functional implants over 10 years was 93.9% [19, 20].

On the other hand, TMC OA causes frequent consultations in outpatient clinics and costs associated with loss of productivity. It leads to substantial economic consequences for the patient, the employer, and society [50].

One of the reasons for the lack of acceptance of the ball and socket prosthesis is the increased cost of the implant without demonstrated superiority over other treatments [17]. Marks et al. reported a healthcare cost of 5.770 euros and a loss of productivity cost of over 5.548 euros for trapeziectomy and ligament arthroplasty [50]. Nowadays, the prosthesis healthcare and productivity costs are unknown.

The aim of the study is to compare the effectiveness of the trapeziectomy with ligament reconstruction arthroplasty with Maïa® prosthesis. Effectiveness indicators should be the relief of pain, functional disability measured by Quick DASH and PWRE, and the improvement of quality of life with EQ-5D-5L.

Up to date, this is the first randomized study that investigates the effectiveness and cost-utility of trapeziectomy and ligament reconstruction arthroplasty and Maïa prosthesis. We expect the findings from this trial to lead to new insights into the surgical approach to TMC OA.

## Trial status

Protocol version #2, June 15, 2020. Registered at Clinicaltrials.gov, identifier: NCT04562753. Currently recruiting. The trial start date was February 2021. The approximate date the recruitment will be completed is February 2026.

### Supplementary Information

**Supplementary Material 1.**

## Data Availability

The final trial data set will be available to the investigators. Results and findings of the study will be released through publications in the scientific literatura and conference presentations.
