# Supplementary material for: Cost-effectiveness and cost-utility of the ball-and-socket trapeziometacarpal prosthesis compared to trapeziectomy and ligament reconstruction: study protocol for a randomized controlled clinical trial
Source: Trials. 2024 Mar 27;25:220. doi: 10.1186/s13063-024-08057-1 (PMC10967180; doi:10.1186/s13063-024-08057-1)
Supplement: Supplementary file 1 — Supplementary Material 1. [file 13063_2024_8057_MOESM1_ESM.pdf]

SPIRIT Checklist for *Trials*

Complete this checklist by entering the page and line numbers where each of the items listed below can be found in your manuscript.

Your manuscript may not currently address all the items on the checklist. Please modify your text to include the missing information. If you are certain that an item does not apply, please state "n/a" and provide a short explanation. **Leaving an item blank or stating "n/a" without an explanation will lead to your manuscript being returned before review.**

Upload your completed checklist as an additional file when you submit to *Trials*. You must reference this additional file in the main text of your protocol submission. The completed SPIRIT figure must be included within the main body of the protocol text and can be downloaded here: http://www.spirit-statement.org/schedule-of-enrolment-interventions-and-assessments/

In your methods section, please state that you used the SPIRIT reporting guidelines, and cite them as:

Chan A-W, Tetzlaff JM, Gøtzsche PC, Altman DG, Mann H, Berlin J, Dickersin K, Hróbjartsson A, Schulz KF, Parulekar WR, Krleža-Jerić K, Laupacis A, Moher D. SPIRIT 2013 Explanation and Elaboration: Guidance for protocols of clinical trials. BMJ. 2013;346:e7586

		Reporting Item	Page and Line Number	Reason if not applicable
**Administrative information**				
Title	#1	Descriptive title identifying the study design, population, interventions, and, if applicable, trial acronym	Page 1<br>Lines: 1,2,3	
Trial registration	#2a	Trial identifier and registry name. If not yet registered, name of intended registry	Page 2<br>Line 38	
Trial registration: data set	#2b	All items from the World Health Organization Trial Registration Data Set	Page 2<br>Line 38	
Protocol version	#3	Date and version identifier	Page 19	

			Line 411 - 413	
Funding	#4	Sources and types of financial, material, and other support	Page 19<br><br>Lines: 415 - 418	
Roles and responsibilities: contributorship	#5a	Names, affiliations, and roles of protocol contributors	Page 19<br><br>Line: 420 - 425	
Roles and responsibilities: sponsor contact information	#5b	Name and contact information for the trial sponsor	Page 19<br><br>Line: 427 - 432	
Roles and responsibilities: sponsor and funder	#5c	Role of study sponsor and funders, if any, in study design; collection, management, analysis, and interpretation of data; writing of the report; and the decision to submit the report for publication, including whether they will have ultimate authority over any of these activities	Page 19<br><br>Line 420 - 425	
Roles and responsibilities: committees	#5d	Composition, roles, and responsibilities of the coordinating centre, steering committee, endpoint adjudication committee, data management team, and other individuals or groups overseeing the trial, if applicable (see Item 21a for data monitoring committee)	n/a	Hospital Son Espases Investigator Committee approved the trial but it is not a overseeing group. The surgery alternative are usual treatment in our regular practice. The randomization and allocation are approved for ethical committee.
**Introduction**				
Background and rationale	#6a	Description of research question and justification for undertaking the trial, including summary of relevant studies (published and unpublished)	Page: 2-4<br><br>Lines: 43-77	

		examining benefits and harms for each intervention		
Background and rationale: choice of comparators	#6b	Explanation for choice of comparators	Page:2-4<br>Lines: 43-77	
Objectives	#7	Specific objectives or hypotheses	Page: 4<br>Lines: 79 - 90	
Trial design	#8	Description of trial design including type of trial (eg, parallel group, crossover, factorial, single group), allocation ratio, and framework (eg, superiority, equivalence, non-inferiority, exploratory)	Page : 5<br>Lines: 92 - 101	
**Methods: Participants, interventions, and outcomes**				
Study setting	#9	Description of study settings (eg, community clinic, academic hospital) and list of countries where data will be collected. Reference to where list of study sites can be obtained	Page: 5<br>Lines: 94-101	
Eligibility criteria	#10	Inclusion and exclusion criteria for participants. If applicable, eligibility criteria for study centres and individuals who will perform the interventions (eg, surgeons, psychotherapists)	Page: 5 - 6<br>Lines 103 - 130	
Interventions: description	#11a	Interventions for each group with sufficient detail to allow replication, including how and when they will be administered	Page:8 - 11<br>Lines: 165 - 249	
Interventions:	#11b	Criteria for discontinuing or modifying allocated	Page: 12	The participant request is the only reason

modifications		interventions for a given trial participant (eg, drug dose change in response to harms, participant request, or improving / worsening disease)	Lines 252 - 258<br>Page: 13<br>Line: 282-284	to discontinuing the trial. Adverse events are part of the regular practice and should be collected in the report form. If the participant must be deallocated it would not change the follow up or our usual treatment.
Interventions: adherance	#11c	Strategies to improve adherence to intervention protocols, and any procedures for monitoring adherence (eg, drug tablet return; laboratory tests)	Page 7 - 8<br>Line: 158 - 163	
Interventions: concomitant care	#11d	Relevant concomitant care and interventions that are permitted or prohibited during the trial	Page 11<br>Line: 232 -249	
Outcomes	#12	Primary, secondary, and other outcomes, including the specific measurement variable (eg, systolic blood pressure), analysis metric (eg, change from baseline, final value, time to event), method of aggregation (eg, median, proportion), and time point for each outcome. Explanation of the clinical relevance of chosen efficacy and harm outcomes is strongly recommended	Page 12 - 13<br>Lines: 257 - 284	
Participant timeline	#13	Time schedule of enrolment, interventions (including any run-ins and washouts), assessments, and visits for participants. A schematic diagram is highly recommended (see Figure)	Page 30 -31<br>Table 2	
Sample size	#14	Estimated number of participants needed to achieve study objectives and how it was	Page 14	

		determined, including clinical and statistical assumptions supporting any sample size calculations	Line: 295 - 301	
Recruitment	#15	Strategies for achieving adequate participant enrolment to reach target sample size	n/a	All patients with thumb base osteoarthritis are evaluated by specialists in the Hand Surgery Unit of the Hospital. They are individually assessed to determine if they meet the inclusion criteria for the clinical trial. If they meet the criteria, the senior surgeon, after explaining the study, offers them all the possibility to enroll in the study. Since it is a single-center trial, patients from other centers or areas cannot be recruited unless the patient themselves requests to be seen and treated at our hospital, or is referred by another specialist.
**Methods: Assignment of interventions (for controlled trials)**				
Allocation: sequence generation	#16a	Method of generating the allocation sequence (eg, computer-generated random numbers), and list of any factors for stratification. To reduce predictability of a random sequence, details of any planned restriction (eg, blocking) should be provided in a separate document that is unavailable to those who enrol participants or assign interventions	Page: 7<br><br>Line: 148 - 157	The OxMaR system, acronym for Oxford Minimization and Randomization, was published as free and open source software in 2014. It works online in a web environment and allows simple randomization and adaptive assignment through minimization. The randomization is adjusted for age and sex.
Allocation concealment mechanism	#16b	Mechanism of implementing the allocation sequence (eg, central telephone; sequentially	Page: 7	

		numbered, opaque, sealed envelopes), describing any steps to conceal the sequence until interventions are assigned	Line: 150-157	
Allocation: implementation	#16c	Who will generate the allocation sequence, who will enrol participants, and who will assign participants to interventions	Page: 7<br><br>Line 151 -158	
Blinding (masking)	#17a	Who will be blinded after assignment to interventions (eg, trial participants, care providers, outcome assessors, data analysts), and how	Page: 7 - 8<br><br>Line: 158 - 163	
Blinding (masking): emergency unblinding	#17b	If blinded, circumstances under which unblinding is permissible, and procedure for revealing a participant's allocated intervention during the trial	n/a	Senior hand surgeon (Dr. Salva-Coll) knows which treatment has been performed, he is not blinded and give the patient the usual treatment in our practice. Dr. Lirola-Palmero, as main investigator, is blinded but is not relevant in the regular follow-up, just to collect data.
**Methods: Data collection, management, and analysis**				
Data collection plan	#18a	Plans for assessment and collection of outcome, baseline, and other trial data, including any related processes to promote data quality (eg, duplicate measurements, training of assessors) and a description of study instruments (eg, questionnaires, laboratory tests) along with their reliability and validity, if known. Reference to where data collection forms can be found, if not in the protocol	N/A	Before of surgery every measure has to be collected in baseline data form. If not collected the could not be operated until it is done.<br><br>Then of surgery every appointment is duplicated, with hand surgeon and main investigator separately.
Data collection plan:	#18b	Plans to promote participant retention and	N/A	If the patient does not attend the

retention		complete follow-up, including list of any outcome data to be collected for participants who discontinue or deviate from intervention protocols		appointment, will be called by phone with a new appointment.<br><br>Then of an appointment they will have the next appointment schedule.
Data management	#19	Plans for data entry, coding, security, and storage, including any related processes to promote data quality (eg, double data entry; range checks for data values). Reference to where details of data management procedures can be found, if not in the protocol	Page 13<br><br>Lines 290 - 291	Data form is locked save in the Hospital. In addition, a computerized database is save.
Statistics: outcomes	#20a	Statistical methods for analysing primary and secondary outcomes. Reference to where other details of the statistical analysis plan can be found, if not in the protocol	Page 14<br><br>Line: 303 - 319	
Statistics: additional analyses	#20b	Methods for any additional analyses (eg, subgroup and adjusted analyses)	Page:15 - 16<br><br>Line: 323 - 356	
Statistics: analysis population and missing data	#20c	Definition of analysis population relating to protocol non-adherence (eg, as randomised analysis), and any statistical methods to handle missing data (eg, multiple imputation)	Page: 14 - 15<br><br>Line 314 - 319	
**Methods: Monitoring**				
Data monitoring: formal committee	#21a	Composition of data monitoring committee (DMC); summary of its role and reporting structure; statement of whether it is independent from the sponsor and competing interests; and reference to where further details about its	N/A	There is not a data monitoring committee. This DMC is not needed because the trial is coordinated and monitored by the PI that is not directly involved in the surgery. Also a statistician,

		charter can be found, if not in the protocol. Alternatively, an explanation of why a DMC is not needed		not directly related to data collection, supervises randomization and assignment
Data monitoring: interim analysis	#21b	Description of any interim analyses and stopping guidelines, including who will have access to these interim results and make the final decision to terminate the trial	N/A	No interim analysis is planned and therefore there are no stopping rules in this clinical trial.
Harms	#22	Plans for collecting, assessing, reporting, and managing solicited and spontaneously reported adverse events and other unintended effects of trial interventions or trial conduct	Page 11- 12<br>Line: 252 - 256	
Auditing	#23	Frequency and procedures for auditing trial conduct, if any, and whether the process will be independent from investigators and the sponsor	N/A	Every year it is necessary to notify the trial evolution to the Ethics Committee of the Balearic Islands. The Orthopedic Spanish Society Foundation, as funder, asks for a evolution report every year.
**Ethics and dissemination**				
Research ethics approval	#24	Plans for seeking research ethics committee / institutional review board (REC / IRB) approval	Page 16<br>Line: 357 - 359	
Protocol amendments	#25	Plans for communicating important protocol modifications (eg, changes to eligibility criteria, outcomes, analyses) to relevant parties (eg, investigators, REC / IRBs, trial participants, trial registries, journals, regulators)	N/A	Every year it is necessary to notify the trial evolution to the Ethics Committee of the Balearic Islands. The Orthopedic Spanish Society Foundation, as funder, asks for a evolution report every year.
Consent or assent	#26a	Who will obtain informed consent or assent from potential trial participants or authorised	Page 7	

		surrogates, and how (see Item 32)	Line: 141 - 143	
Consent or assent: ancillary studies	#26b	Additional consent provisions for collection and use of participant data and biological specimens in ancillary studies, if applicable	N/A	Not applicable, no samples collected.
Confidentiality	#27	How personal information about potential and enrolled participants will be collected, shared, and maintained in order to protect confidentiality before, during, and after the trial	Page 7<br>Line: 150 - 157	
Declaration of interests	#28	Financial and other competing interests for principal investigators for the overall trial and each study site	N/A	The autors declare that they have no competing interests.
Data access	#29	Statement of who will have access to the final trial dataset, and disclosure of contractual agreements that limit such access for investigators	N/A	Every investigator has access to the dataset, changes has to be approved by Dr. Lirola-Palmero as main investigator.
Ancillary and post trial care	#30	Provisions, if any, for ancillary and post-trial care, and for compensation to those who suffer harm from trial participation	N/A	Both treatments are part of our regular practice, like patients who are not enrolled in the trial. The patients sign the consent approved by Spanish Society Hand Surgery, approved by our Hospital.
Dissemination policy: trial results	#31a	Plans for investigators and sponsor to communicate trial results to participants, healthcare professionals, the public, and other relevant groups (eg, via publication, reporting in results databases, or other data sharing arrangements), including any publication restrictions	Page 13<br>Line 292 -293	

Dissemination policy: authorship	#31b	Authorship eligibility guidelines and any intended use of professional writers	N/A	This trial is the Dr. Lirola-Palmero's PhD thesis. Every publication will be directed by Dr. Lirola-Palmero and supported by Dr. Salva-Coll, Dr. Yañez-Juan and Dr. Sanchez-Iriso.
Dissemination policy: reproducible research	#31c	Plans, if any, for granting public access to the full protocol, participant-level dataset, and statistical code	N/A	The patients receive an information sheet the same day they sign the informed consent. This information sheet includes the summary version of the protocol (added in #32)
**Appendices**				
Informed consent materials	#32	Model consent form and other related documentation given to participants and authorised surrogates	Added	Added
Biological specimens	#33	Plans for collection, laboratory evaluation, and storage of biological specimens for genetic or molecular analysis in the current trial and for future use in ancillary studies, if applicable	N/A	Not applicable, no samples collected.

It is strongly recommended that this checklist be read in conjunction with the SPIRIT 2013 Explanation & Elaboration for important clarification on the items. Amendments to the protocol should be tracked and dated. The SPIRIT checklist is copyrighted by the SPIRIT Group under the Creative Commons "Attribution-NonCommercial-NoDerivs 3.0 Unported" license. This checklist can be completed online using https://www.goodreports.org/, a tool made by the EQUATOR Network in collaboration with Penelope.ai
